# The Effectiveness of Nucleoside Analogues in Chronic Hepatitis B Patients Unresponsive to Interferon Therapy: Our Clinical Trials for One Year

**Published:** 2010-12-01

**Authors:** Sukran Kose, Melda Turken, Gulsun Cavdar, Gulgun Akkoclu

**Affiliations:** 1 Tepecik Research and Training Hospital, Clinic of Clinical Microbiology and Infectious Diseases, Tepecik, Izmir, Turkey

**Keywords:** Hepatitis B Virus, nucleoside analogues, interferon therapy

## Abstract

**Background and Aims:**

We aimed to evaluate the effectiveness of nucleoside analogues such as Lamivudine, Adefovir,Entacavir, and Tenofovir in patients with chronic hepatitis B who failed to respond to interferon therapy and relapsed.

**Materials and Methods:**

We followed a total of 73 patients with hepatitis B in the hepatitis outpatient clinic in our hospital. The patients subsequently received nucleoside analogues therapy and their treatment data were evaluated retrospectively. The biochemical and virological response rates were evaluated at 3 and 12 months, and we compared these results with the results of treatment-naive patients.

**Results:**

There were 29 (39.7%) HbeAg-positive and 44 (60.3%) HbeAg-negative patients, and their mean age was 35.8 (±13.4) years. Of these patients, 33, 18, 13 and 9 received Entacavir, Tenofovir, Lamivudine, and Adefovir treatment,respectively. In HbeAg-negative patients, at 3 months the biochemical and virological response (early response) rates were observed to be 91% and 98%), and at 12 months the two rates were 93% and 73%, respectively. In HbeAg-positive patients, the biochemical and virological response rates at 3 months were 83% and 97%, and the rates at 12 months were 90% and 48%, respectively.

**Conclusions:**

In CHB therapy with treatment-resistent patients, nucleoside analogues may be preferable. There are disadvantages to nucleoside analogues, such as a risk of developing resistance during therapy, reduced HBeAg seroconversion compared to interferons, and the therapy’s ambiguous duration. In our study, in HbeAg-negative patients who received nucleoside analogues, a lower biochemical response rate was detected in patients with 1 year of Lamivudine therapy compared to other therapies. For HbeAg-positive patients, the virological response rate was higher in 1 year of Tenofovir therapy than with other therapies.

## Introduction

Chronic infection with hepatitis B virus (HBV) is a major global health problem, affecting more than 400 million people worldwide. The aim of chronic hepatitis B treatment is to stop HBV replication and prevent the development of cirrhosis, liver insufficiency, and hepatocelluler carcinoma [1]. In chronic hepatitis B (CHB) patients, the response criteria are testing positive for HBeAg positive, having HBV DNA that become negative (virological response), experiencing HbeAg or anti-HBeAg seroconversion (serological response), having ALT that turn normal (biochemical response), or recovering histologically [2]. In HBeAg-negative patients, the response criteria are the normalizing of ALT levels and having negative or undetectible HBV DNA levels. Sustained response is the persistence of a response to therapy for 6 months after the therapy is discontinued. In patients for whom therapy was initiated, the absence of virological, serologic, or biochemical responses implies unsuccessful therapy; however, for patients who respond at the end of therapy, HBV DNA becoming positive, the elevation of ALT, or reversion (repositivity of HBeAg) are considered relapses. HBV DNA that were negative during therapy but become positive afterwards is a sign of viral "breakthrough" [2]. Interferons have been used in the treatment of chronic hepatitis B for many years. In early studies; various therapies were provided to patients with previous treatment failures, with variable success rates. Therapies for patients with failed interferon therapy include retreatment with standard interferon, a combination of interferon and Lamivudine, a combination of hepatitis B surface antigen vaccination and interferon, pegylated interferon, a combination of  pegylated interferon and Lamivudine, Lamivudine by itself, and Adefovir. Unfortunately, the response rate with  those treatment methods  was not high [3]. Today, interferons (conventional and pegylated), nucleoside analogues (Lamivudinee, Entacavir, telbuvidin, and emstrisitabin), and nucleotide analogues (Adefovir and Tenofovir) are used in CHB treatment. Among these treatment options, conventional Interferons (IFN), Pegylated Interferons (peg-IFN), Lamivudinee (LAM), Adefovir (ADV), Entacavir (ETV), and Tenofovir (TDF) are the drugs available and approved for use in Turkey. In the literature, there is no study that compares the effectiveness of nucleozide-analogue therapies in cases unresponsive to IFN.In the present study, we evaluated the annual effectiveness of Lamivudinee, Adefovir, Entacavir, and Tenofovir in CHB patients who failed to respond to conventional or Pegylated IFN therapy and developed relapses, and we compared our results with treatment-naive patients.

## Materials And Methods

We retrospectively evaluated a total of 73 patients with hepatitis B in the hepatitis  outpatient clinic affiliated with the Department of Infectious Diseases and Clinical Microbiology of the Izmir Tepecik Training and Research Hospital who previously failed to respond to classic or peg-IFN therapy or developed relapses and were subsequently treated with nucleoside analogues. Of the the experimental group, 33 (45.2%) received Entacavir, 18 (24.6%) received Tenofovir, 13 (17.8%) received Lamivudinee, and 9 (12.3%) received Adefovir therapy, respectively. None of the patients who were included in the study had previously received another nucleoside analogues therapy. We composed the control group with naive patients who did not recieve IFN or other antiviral agents before treatment with nucleozide analogues. Of the control group, 15 (34,8%) received Entacavir, 21 (%48,8%) received Tenofovir, 5 (11,6%) received Lamivudinee, and 2 (4,6%) received Adefovir. We compared the experimental group with the control group. Patients' liver function, HBeAg, anti-HBe, and HBV DNA levels were monitored for 3 months. During treatment, biochemical and virological response rates were evaluated at 3 and 12 months. For this study, we used the VHSD Guidelines for Viral Hepatitis Diagnosis and Treatment (2009) to define biochemical response  as the normalization of ALT (<35 IU/mL), serological response as HbeAg or anti-HBe seroconversion; and virological response as at least a 2-logarithmic decrease in HBV DNA at 3 months and HBV DNA reaching negative or undetectible levels by 12 months. We classifed patients as unresponsive to treatment if they did not fit these criteria. First-year Lamivudinee and Adefovir resistance were determined. Statistical analyses were performed using Fisher's chi-square tests, McNemar's test, and  t-tests. This study was conducted with the approval of the ethics committee of Tepecik Research and Educational Hospital.

## Results

In the experimental group, 27 (37%) and 46 (63%) of the total 73 CHB patients were female and male, respectively. The patients' mean age was 35.8 years (±13.4). There were 29 (39.7%) HBeAg-positive and 44 (60.3%) HBeAg-negative patients in the group. Previously, 36 patients (49.3%) had received classic IFN therapy and 37 (50.7%) received peg-IFN therapy due to CHB. In the control group, 21 (50%) and 21 (50%) of the total 42 CHB patients were female and male, respectively. The patients' mean age was 34.8 years (±11.9). There were 19 (45.2%) HBeAg-positive and 23 (54.8%) HBeAg-negative patients in the group. The biochemical and virological responses at the end of 3 and 12 months are given in tables 1 and 2 according to the therapies given to the patients in the experimental group and the control group who were HBeAg negative and positive, respectively.In HbeAg-negative patients, whereas a significant difference was not found between therapies in terms of a biochemical response at 3 months or a virological response at 3 or 12 months (P>0.05), the biochemical response at 12 months was significantly lower in the Lamivudinee group compared to the Adefovir, Tenofovir, and Entacavir groups (P≤0.05). The biochemical response at 3 months in HBeAg-negative patients who recieved Tenofovir and Adefovir in the experimental group were signigficantly higher than the control group (P≤0.05). The biochemical response at 12 months in patients who recieved Adefovir in the experimental group was signigficantly higher than the control group (P≤0.05). In HbeAg-positive patients, whereas there were no significant differences between therapies in terms of the biochemical response at 3 months; a virological response at 3 or 12 months; or virological and serological responses at 12 monhts (P>0.05), an elevated virological response was observed in the Tenofovir group at 1 year compared to the Lamivudinee, Adefovir, and Entacavir groups (P≤0.05). Only 1 patient who used Adefovir in the HBeAg-positive group did not have a virological response by 12 months. There was no significant difference statistically (P>0.05). Virological response at 12th month in HBeAg positive patients who recieved Tenofovir in experimental group were signigficantly higher than control group (P≤0.05). HbeAg seroconversion in HBeAg positive patients who recieved Tenofovir and Entacavir in experimental group were signigficantly higher than control group (P≤0.05). In the experimental and control groups, the mean ALT, HBV DNA, and HAI levels at baseline, 3 months, and 12 months of treatment are shown in table 3.The mean ALT, HBV DNA, and HAI levels at the beginning of treatment were not significantly different between the experimental and control groups (P>0.05). In the HBeAg-positive patients, whereas the mean ALT levels were not significantly different between the experimental and control groups at 3 or 12 months (P>0.05), the mean HBV DNA levels were found to be significantly lower in the experimental group (P≤0.05).  In the HBeAg-negative patients, whereas the mean HBV DNA levels were not significantly different between the experimental and control groups at both timepionts (P>0.05) at 3 or 12 months, the mean ALT levels were significantly lower in the experimental group at both timepoints (P≤0.05). In the experimental and control groups, the comparisons of the bichemical, virological, and serological responses and confidence intervals (CI 95%) are shown in table 4.YMDD and YVDD mutations developed in 2 (11.7 %) Lamivudine-treated patients. Entakavir and Tenofovir resistance were not examined.

**Table 1:** The percentage of response to antiviral therapy in the patients with HBeAg negative and unpresponsive to IFN

**Drug**	**Experimental group**					**Control group**				
****-	**n**	**Virological response**		**Virological response**		**n**	**Virological response**		**Biochemical response**	
****-		**3^rd^ mon (%)**	**12^th^ mon (%)**	**3^rd ^mon (%)**	**12^th ^mon (%)**		**3^rd^ mon (%)**	**12^th^ mon (%)**	**3^rd ^mon (%)**	**12^th^ mon (%)**
**Lamivudine**	5	80	60	100	80	3	66.7	33.3	100	100
**Adefovir**	8	87.5	50	100	100	1	100	0	0	0
**Tenofovir**	12	100	100	83.3	100	13	100	76.9	30.8	76.9
**Entacavir**	19	94.7	68.4	89.5	100	6	100	83.3	66.7	100
**Total**	44	93.2	72.7	90.9	97.7	23	95.6	82.6	47.8	82.6

**Table 2:** The percentage of response against antiviral therapy in the patients with HBeAg positive and unpresponsive to IFN

**Drug**	**Experimental group**					**Control group**				
	**n**	**Virological response**		**Biochemical response**		**n**	**Virological response**		**Biochemical response**	
		**3^rd^ mon (%)**	**12^th^ mon (%)**	**3^rd ^mon (%)**	**12^th^ mon (%)**	**-**	**3^rd^ mon (%)**	**12^th^ mon (%)**	**3^rd ^mon (%)**	**12^th^ mon (%)**
**Lamivudine**	8	87.5	25	62.5	87.5	2	66.7	50	100	100
**Adefovir**	1	100	2	100	100	0	0	0	0	0
**Tenofovir**	6	100	100	83.3	100	8	100	37.5	62.5	87.5
**Entacavir**	14	85.7	42.9	82.9	100	9	100	66.7	66.7	88.9
**Total**	29	89.7	48.3	82.8	96.6	19	100	52.6	68.4	89.4

**Table 3:** Comparison of mean levels of ALT, HBV DNA, and HAI at baseline, 3 months, and 12 months of treatment

****		**Experimental group**		**Control group**	
****		**HBeAg (+)**	**HBeAg (-)**	**HBeAg (+)**	**HBeAg (-)**
**The begining**	ALT (IU/L)	84.1 (± 56.3)	131.7 (100.2)	122.1 (± 129.7)	99.7 (± 63.7)
	HBVDNA (Copy/ml)	7x10 ^7^ (± 5.3x10 ^7^)	1.8x10 ^7^(± 3.5x10 ^7^)	8.3x10^ 7^(± 3.4x10 ^7^)	7.5x10 ^7^(± 1.6x10 ^8^)
	HAİ	8.4 (± 3.6)	9.5 (± 3.9)	9.4 (± 3.3)	8.4 (± 3.8)
**3^rd ^mon**	ALT (IU/L)	32.7 (± 17.6)	27.4 (± 10.7)	46.9 (± 50.4)	52.8 (± 38.5)
	HBVDNA (Copy/ml)	6.4x10 ^3^(± 6.8x10^ 3^)	2.5x10 ^3^(± 2.3x10 ^3^)	6.7x10^ 4^(± 9.5x10 ^4^)	2.5x10 ^4^(± 1.2x10 ^7^)
**12^th^ mon**	ALT (IU/L)	26.3 (± 6.8)	24.0 (± 4.9)	25.3 (± 7.3)	33.2 (± 17.5)
	HBVDNA (Copy/ml)	2.3x10 ^2^(± 4.7x10 ^2^)	1.2x10 ^2^(± 3.3x10 ^2^)	5.9x10 ^2^(± 7.1x10 ^2^)	2.1x10 ^3^(± 9.5x10 ^3^)

**Table 4:** Comparison of bichemical, virological, and serological responses and confidence intervals.

**-**	**HBeAg status**	**Experimental group**	**Control group**	**OR**	**CI (95%)**
**3^rd^ mo VR(%)**	+	89.7	100	1.115	0.986-1.262
	-	93.2	95.7	0.621	0.061-6.332
**12^th ^mo VR(%)**	+	48.3	52.6	0.840	0.264-2.675
	-	72.7	69.6	1.167	0.385-3.535
**3^rd ^mo BR(%)**	+	82.8	68.4	2.215	0.566-8.678
	-	90.6	47.8	10.9	2.933-40.580
**12^th ^mo BR(%)**	+	96.6	89.5	3.294	0.277-39.138
	-	97.7	82.6	9.053	0.948-86.479
**12^th ^mo SR (%)**	+	13.8	0	-	-

## Discussion

In the previous studies, for patients who were unresponsive to IFN therapy, IFN therapy was retried, but in these studies the number of subjects was generally small. Repeated IFN therapy in patients who fail to respond may cause HBeAg seroconversion, and the patient's HBV DNA can became negative. In HBeAg-negative patients who fail to respond to IFN therapy, HBV DNA negativity can be achieved in up to 22% of patients by repeating the IFN treatment [2][4][5]. In cases unresponsive to IFN, IFN + Lamivudine has been shown to be superior to IFN monotherapy in terms of HBV DNA becoming negative and HBeAg seroconversion [2][6][7][8]. In HBeAg-positive patients who fail to respond to IFN therapy, HBV DNA negativity can be achieved in up to 29.4% of patients with Lamivudine therapy. In a study carried out with HBeAg-positive children who failed to respond to IFN therapy, HBV DNA negativity was obtained in 44% and HBeAg seroconversion in 5% for patients through Lamivudine therapy [9]. During this study, long-term Lamivudine results were evaluated. Four years after starting Lamivudine treatment, most children from this study were off Lamivudine, mainly because of lack of compliance and poor HBe seroconversion [10]. Again, in another study conducted on pediatric group with a one-year Lamivudine therapy, an HBeAg/antiHBe seroconversion rate of was detected in 10% of cases unresponsive to IFN [11]. In some studies, the therapy responses obtained by Lamivudine in patients who are unresponsive to IFN were similar to those obtained in naïve patients. Schiff et al. compared Lamivudine and Lamivudine+IFN therapy in 238 HBeAg-positive patients who were unresponsive to IFN therapy with a placebo control group. A higher ALT-normalization was found in the Lamivudine monotherapy group (44%) compared to the combination therapy group (18%) and the placebo group (15%). Loss of HBeAg was established as 33%, 23%, and 13%, respectively. Histologic recovery was determined to be 52% in the Lamivudine group, 32% in the combination group, and 25% in the placebo group [8].  In a study carried out in Turkey, 2 years of Lamivudine monotherapy and Lamivudine+IFN combination therapy were compared in HBeAg-negative CHB patients who were unresponsive to IFN. The therapy response was 29.2% in those receiving Lamivudine monotherapy and 19% in those receiving Lamivudine+IFN combination therapy. The YMDD mutation rate at the end of 2 years was found to be 59.2% in the first group and 62.5% in the second group [12]. These findings suggest that the use of Lamivudine therapy in patients who fail to respond to IFN therapy would be helpful.  In two different studies, Adefovir was attempted in 123 HbeAg-positive and 48 HbeAg-negative patients who received IFN previously and failed to respond. An HBeAg seroconversion rate similar to the response rates in naïve patients was found. After administration of Adefovir, the HBeAg seroconversion rates in patients treated previously with interferon were similar to those in naïve patients [13][14]. Entakavir has been tried on cases who received IFN previously but not Lamivudine, and it has been shown to be effective [13]. In CHB therapy for patients who fail to respond or develop relapses after interferon treatment, nucleous(t)id analogues may be preferable. There are disadvantages, such as a risk of developing resistance during therapy, reduced HBeAg seroconversion compared to interferons, and the ambiguity of the therapy's duration. In our study, whereas the 1-year response rate was good (72.7%) in HBeAg-negative patients who failed to respond to interferon therapy and received nucleous(t)id analogues, reduced serologic (13.8%) and virological response (48%) rates were observed in HBeAg-positive patients. However, antiviral agents are still an important choice as an alternative drug in CHB therapy for patients who are unresponsive to IFN. In the literature, no previous study had compared the effectiveness of nucleozid-analogues therapies in cases unresponsive to IFN. In our study, we compared the 1-year effectiveness of lamuvudin, Adefovir, entakavir, and Tenofovir. After 1 year of therapy, a significantly lower biochemical response rate was found in the Lamivudine group compared to other groups, and the virological response rate was significantly higher in the Tenofovir group than the other groups. As a result, our contention is that this study can inform nucleozid-analogues therapy for patients who fail to respond to IFN and develop relapses.  Finally, much more studies are needed in this regard.
